# Genome Sequence of Feline Papillomavirus Strain P20 Assembled from Metagenomic Data from the Skin of a House Cat Owner

**DOI:** 10.1128/mra.01070-21

**Published:** 2022-07-05

**Authors:** Ema H. Graham, Michael S. Adamowicz, Peter C. Angeletti, Jennifer L. Clarke, Samodha C. Fernando, Joshua R. Herr

**Affiliations:** a Ph.D. Program in Complex Biosystems, University of Nebraska, Lincoln, Nebraska, USA; b College of Agricultural Sciences and Natural Resources, University of Nebraska, Lincoln, Nebraska, USA; c Nebraska Center for Virology, University of Nebraska, Lincoln, Nebraska, USA; d School of Biological Sciences, University of Nebraska, Lincoln, Nebraska, USA; e Department of Statistics, University of Nebraska, Lincoln, Nebraska, USA; f Food Science and Technology Department, University of Nebraska, Lincoln, Nebraska, USA; g Department of Animal Science, University of Nebraska, Lincoln, Nebraska, USA; h Department of Plant Pathology, University of Nebraska, Lincoln, Nebraska, USA; i Center for Plant Science Innovation, University of Nebraska, Lincoln, Nebraska, USA; KU Leuven

## Abstract

A feline papillomavirus genome was assembled from metagenomic sequencing data collected from the skin of a house cat owner. The circular genome of strain P20 is 8,069 bp in length, has a GC content of 54.38%, and displays genome organization typical of feline papillomaviruses. The genome exhibits approximately 75% sequence similarity to other feline papillomavirus genomes.

## ANNOUNCEMENT

Papillomaviruses broadly infect mammals, reptiles, and birds (1). Conservation of the major capsid protein L1 suggests that the evolution of papillomaviruses mirrors the phylogeny of their hosts (2). Recent studies have reported on the discovery and diversity of papillomaviruses associated with domestic and wild cats (3). Seven types of feline papillomaviruses, which may or may not produce both skin and oral squamous cell carcinomas in house cats, are currently recognized (4).

We previously studied the diversity and stability of DNA viruses on human skin by sampling three anatomical locations, i.e., left hand, right hand, and scalp, longitudinally over a 6-month period for 43 human subjects (5). Briefly, samples were collected using a tandem dry and wet swab technique. Swabs were then saturated in 1× phosphate-buffered saline (PBS) and centrifuged at 20,000 × *g*. The sample eluent was run through a 0.22-μm filter to remove cellular and bacterial contaminants, and the resulting filtrate was used for viral DNA extraction using the QIAmp ultrasensitive virus kit (Qiagen, Hilden, Germany) and whole-genome amplification (WGA) using the TruePrime WGA kit (Syngen Biotechnology, Inc., Taipei City, Taiwan), each according to the manufacturer's protocol. The resulting DNA was sheared to 600 bp prior to library preparation using the NEBNext Ultra II library preparation kit (New England Biolabs, Ipswich, MA, USA) according to the manufacturer’s protocol. The libraries were then sequenced as 150-bp paired-end reads on the HiSeq 2500 platform (Illumina, Inc., San Diego, CA, USA). We identified one participant with a metagenome that consistently showed the presence of a feline papillomavirus, who self-identified as being an owner of a domesticated house cat (5).

To investigate further, 15 metagenome samples from that specific human individual were mapped to a *Papillomaviridae* reference database compiled from NCBI using BBMap v38.94, with a minimum match of 95% base pair similarity and a k value of 13 (6). Mapped reads were processed using khmer v2.0.0 (7) to remove singletons and overly abundant reads. Filtered papillomavirus reads were *de novo* assembled using MEGAHIT v1.2.8 (8), and assembly quality was assessed using QUAST v5.0.2 (9). Read coverage for the assembly was an average of 295.18 reads per base, with a standard deviation of 46.61 reads per base. A single contig was evaluated for completeness using CheckV v0.7.0 (10) and nucleotide-based classification tools, such as Kraken2 v2.0.8-beta (11), Demovir (https://github.com/feargalr/Demovir), and BLASTn (with a >10% query coverage cutoff value) (12), as well as the protein-coding classification tool Kaiju v1.7 (13). All tools were run with default parameters unless otherwise specified.

**FIG 1:**
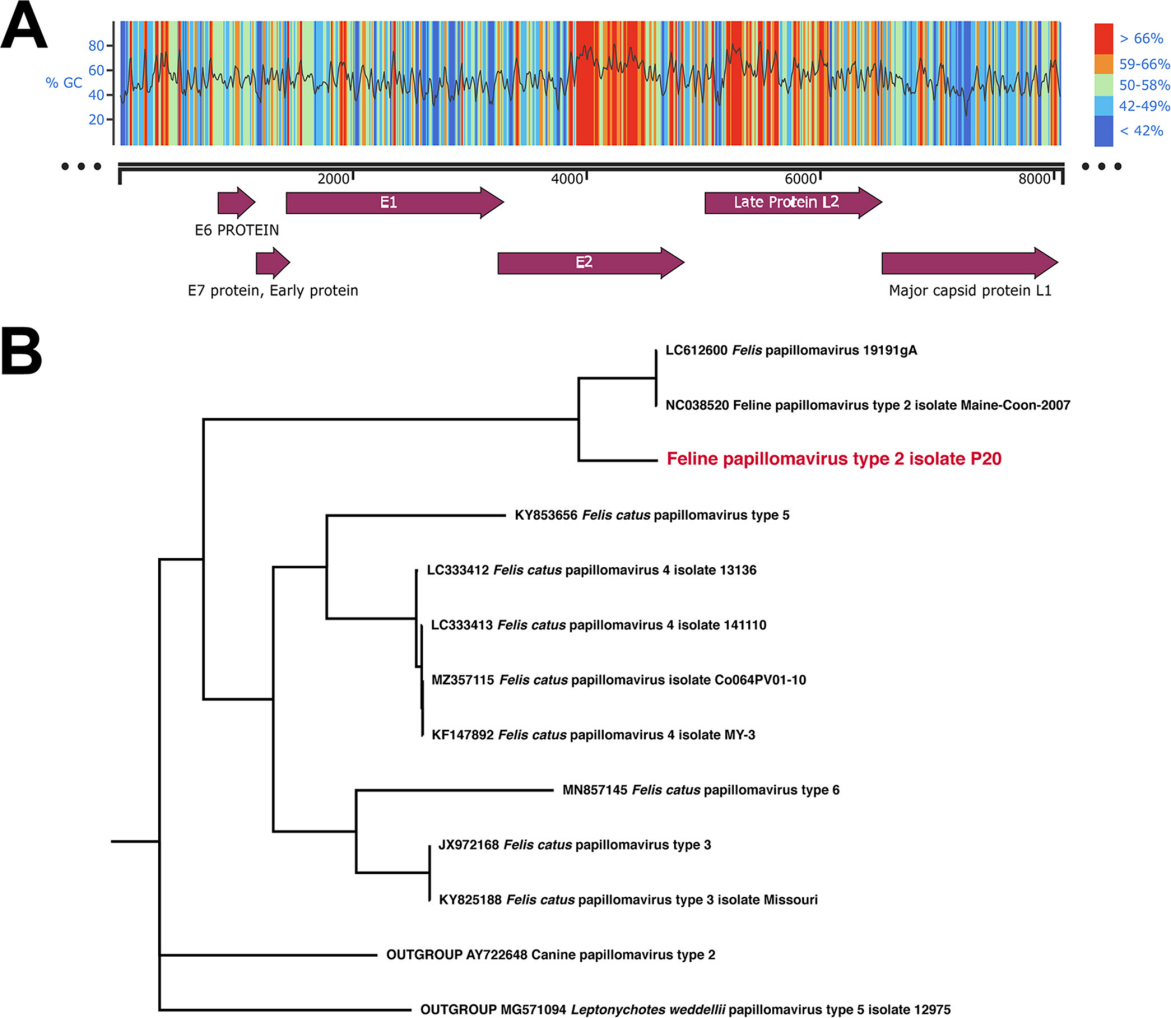
(A) Linear genome map (8,069 bp in length) of the feline papillomavirus type 2-like virus. The top portion shows the GC contents for regions of the circular genome. Higher GC contents are shown in warmer colors (red), and lower GC contents are shown in cooler colors (blue). The locations of the six gene regions in the genome are shown below, with open reading frame annotations (purple arrows) for the E6 protein, E7 protein, E1 protein, E2 protein, late protein L2, and major capsid protein L1. (B) Phylogenetic tree of the placement of the feline type 2 papillomavirus identified in this study along with nine other feline papillomaviruses. Identifiers on the phylogenetic tree correspond to NCBI Genbank accession numbers. The tree was rooted with canine papillomavirus type 2 and Leptonychotes weddelii (Weddell seal) papilloma virus type 5.

We assembled a complete circular feline papillomavirus genome, identified as strain P20, that was 8,069 bp in length and exhibited a GC content of 54.38%. Six open reading frames, including the E6 protein, E7 protein, E1 protein, E2 protein, late protein L2, and major capsid protein L1, which are shared among many animal papillomaviruses (1), were annotated with Prokka v1.14.5 (14) using the embedded viral annotation database and Cenote-Taker 2 v2.1 (15) (Fig. 1) (visualized with SnapGene; GSL Biotech LLC, San Diego, CA). Using BLASTn, our genome assembly showed the greatest similarity (with an average of 70.3% shared amino acids) to feline papillomavirus type 2 (family *Papillomaviridae*, genus *Dyothetapapillomavirus*) isolated from the skin of a domestic Maine Coon house cat in 2007 (GenBank accession number NC_038520).

To determine the phylogenetic placement of strain P20, we downloaded all feline papillomavirus genomes available in the NCBI database (along with two outgroups from closely related hosts, namely, domestic dog and Weddell seal) and aligned the six shared annotated genes using MUSCLE v3.8.1551 (16). After removal of uninformative amino acid regions using Gblocks v0.91b (17), a phylogenetic tree (Fig. 1) was generated using IQ-TREE v1.6.12 (18) with the LG+F+I+G4 substitution model, which was determined to be optimal by ModelFinder v1.5.4 (19). This phylogeny, visualized with FigTree v1.4.4 (https://github.com/rambaut/figtree), placed our genome in proximity to other feline papilloma type 2 strains.

### Data availability.

All raw sequencing data have been deposited in the NCBI Sequence Read Archive (SRA) under the accession number PRJNA754140. Within that BioProject, the 15 sequencing files used in this study had the following accession numbers: SRS9770471, SRS9770472, SRS9770476, SRS9770495, SRS9770500, SRS9770501, SRS9770587, SRS9770588, SRS9770589, SRS9770614, SRS9770616, SRS9770617, SRS9770642, SRS9770643, and SRS9770644. The complete feline papillomavirus P20 genome has been submitted to NCBI GenBank with the accession number OL310516. All metadata, sequences, annotation files, and scripts used here are publicly available and archived at https://github.com/HerrLab/Graham_2021_feline_papilloma_P20.
